# Comprehensive Cardiovascular magnetic resonance of myocardial mechanics in mice using three-dimensional cine DENSE

**DOI:** 10.1186/1532-429X-13-83

**Published:** 2011-12-30

**Authors:** Xiaodong Zhong, Lauren B Gibberman, Bruce S Spottiswoode, Andrew D Gilliam, Craig H Meyer, Brent A French, Frederick H Epstein

**Affiliations:** 1 MR R&D Collaborations, Siemens Healthcare, Atlanta, USA; 2 Radiology Department, University of Virginia, Charlottesville, USA; 3 MRC/UCT Medical Imaging Research Unit, University of Cape Town, Cape Town, South Africa; 4 R&D Department, A.D. Gilliam Consulting, Providence, USA; 5 Biomedical Engineering Department, University of Virginia, Charlottesville, USA

## Abstract

**Background:**

Quantitative noninvasive imaging of myocardial mechanics in mice enables studies of the roles of individual genes in cardiac function. We sought to develop comprehensive three-dimensional methods for imaging myocardial mechanics in mice.

**Methods:**

A 3D cine DENSE pulse sequence was implemented on a 7T small-bore scanner. The sequence used three-point phase cycling for artifact suppression and a stack-of-spirals *k*-space trajectory for efficient data acquisition. A semi-automatic 2D method was adapted for 3D image segmentation, and automated 3D methods to calculate strain, twist, and torsion were employed. A scan protocol that covered the majority of the left ventricle in a scan time of less than 25 minutes was developed, and seven healthy C57Bl/6 mice were studied.

**Results:**

Using these methods, multiphase normal and shear strains were measured, as were myocardial twist and torsion. Peak end-systolic values for the normal strains at the mid-ventricular level were 0.29 ± 0.17, -0.13 ± 0.03, and -0.18 ± 0.14 for *E_rr_*, *E_cc_*, and *E_ll_*, respectively. Peak end-systolic values for the shear strains were 0.00 ± 0.08, 0.04 ± 0.12, and 0.03 ± 0.07 for *E_rc_*, *E_rl_*, and *E_cl_*, respectively. The peak end-systolic normalized torsion was 5.6 ± 0.9°.

**Conclusions:**

Using a 3D cine DENSE sequence tailored for cardiac imaging in mice at 7 T, a comprehensive assessment of 3D myocardial mechanics can be achieved with a scan time of less than 25 minutes and an image analysis time of approximately 1 hour.

## Background

The application of noninvasive cardiac imaging to genetically-engineered mice is widely used to evaluate the roles of individual genes in cardiac function. These methods have been used to elucidate the roles of genes in normal function and the effects of genes and therapies in cardiac dysfunction [1-5], with the latter typically employing mouse models of myocardial infarction [6,7] or heart failure [8,9]. Cardiovascular magnetic resonance (CMR) and echocardiography are both commonly used for these studies [1,2], however CMR is generally considered to be the more accurate modality. Using cine imaging, left ventricular (LV) volume, stroke volume, ejection fraction, and wall thickening can be quantified. In addition, using tissue tracking methods, myocardial strain, twist, and torsion (collectively termed cardiac mechanics) can be measured, which can yield insights into contractile function that are not provided by conventional cine imaging.

A number of different CMR tissue tracking methods may be used to assess cardiac mechanics, including myocardial tagging [10,11], harmonic phase analysis (HARP) [12], velocity encoded phase contrast (PC) [13,14], and displacement encoding with stimulated echoes (DENSE) [15,16]. Furthermore, all of these methods are applicable to both humans [10-17] and mice [18-33]. Among these techniques, tagging is the most widely used, but has the significant disadvantage that image analysis is cumbersome and time consuming. HARP simplifies tag analysis, but does so at the expense of reduced spatial resolution. PC imaging can provide pixel-wise velocity data and direct extraction of velocity values from phase images of the velocity-encoded data. However, extra integral calculation is required to obtain displacement information, and tracking error may accumulate during this procedure [14]. DENSE provides the advantages of high spatial resolution, rapid image analysis, and high displacement accuracy. While some prior tagging studies in mice have been fairly comprehensive in terms of imaging coverage and analysis of three-dimensional (3D) mechanics [20,23], prior DENSE studies have not been fully comprehensive in these regards. We recently reported on the development of a cine DENSE method that quantifies 3D mechanics throughout the entire LV [34]. In that report, 3D cine DENSE was applied to imaging humans using a 1.5 T scanner. In the present study we adapted these methods for imaging mice using a high-field, small-bore scanner, and used them to comprehensively assess the 3D mechanics of the mouse LV.

## Methods

### 3D cine DENSE Pulse Sequence and Image Reconstruction

**Figure 1:**
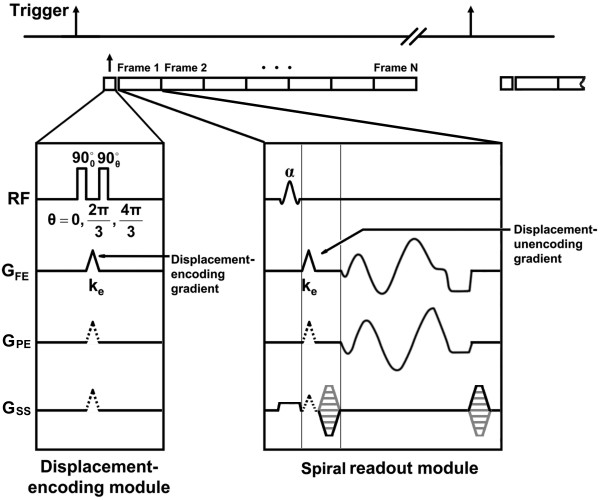
**Timing diagram for the 3D cine DENSE pulse sequence**. Timing diagram for the 3D cine DENSE pulse sequence. Upon detection of an ECG trigger, a displacement-encoding module is played out. Phase cycling of the second radiofrequency pulse is used for artifact suppression. The displacement-encoding module is followed by successive multiphase applications of a readout module, which includes a displacement unencoding gradient. A stack-of-spirals *k*-space trajectory is used to achieve efficient data sampling. For mouse heart imaging at 7T, a relatively short spiral readout is used to reduce off-resonance-induced blurring.

The 3D cine DENSE method recently developed for imaging the human LV on a 1.5 T scanner [34] was modified for imaging the mouse LV at a field strength of 7 T. As shown in Figure 1, immediately following R-wave trigger detection, a displacement encoding module consisting of RF and gradient pulses is applied, which stores position-encoded magnetization along the longitudinal axis. The time required to apply the displacement-encoding pulses is 2 ms, which is very short relative to the RR interval, and it can be assumed that no cardiac motion occurs during this period. Also, three-point phase cycling of the second displacement-encoding RF pulse is employed for suppression of artifact-generating echoes [35]. The initial displacement-encoding module was followed by successive (multiphase) applications of a readout module, which employed an RF excitation pulse, a displacement unencoding gradient, and an interleaved and segmented stack-of-spirals trajectory to sample the 3D *k*-space. A spiral trajectory was chosen to achieve a short echo time (TE) and a time-efficient sampling of *k*-space [36]. Displacement-encoding gradients were designed using the shortest possible time, and, when applicable, unencoding gradients were combined with phase-encoding gradients in the slice-select direction to minimize TE. In addition to ECG triggering, respiratory gating was also used to reduce motion artifact. Multiphase data were acquired throughout at least 90% of the RR interval, followed by a delay of at least 250 ms to allow for T1 relaxation. After the delay, the sequence execution resumed after the next detected trigger. Also, a B0 field map was acquired just prior to acquisition of DENSE data to support off-resonance correction during image reconstruction (deblurring). Field map scanning included two 3D acquisitions with different TEs, where each used a single spiral interleave per 3D partition.

DENSE magnitude and phase images were reconstructed online. First, deblurring of the 3D stack-of-spirals dataset was performed as described previously [37]. Briefly, the time signals for both the map and image data were Fourier transformed along the partition direction to form two-dimensional (2D) data sets localized along that direction, and then linear inhomogeneity compensation was performed independently for each slice. Cancellation of interference from artifact-generating echoes and isolation of the displacement-encoded stimulated echo were achieved by combination of the phase cycled datasets as previously described [35]. Subtraction of the background phase was performed to obtain phase images encoded for displacement in the *x*, *y *and *z *directions. The corresponding overall magnitude image at each cardiac phase was also calculated using the square root of the sum of the squares of the stimulated echo for all encoding directions.

### Computation of Cardiac Mechanics from 3D Cine DENSE Images

The computation of myocardial strain, twist, and torsion was performed offline in MATLAB (Mathworks Inc., Natick, MA, United States), and included segmentation of the myocardium, phase unwrapping and tissue tracking, and subsequent calculation of specific mechanics parameters. The myocardium was segmented partition-by-partition using a semi-automatic motion-guided segmentation method where manual delineation of the endocardial and epicardial contours is performed at one cardiac phase, and then these contours are automatically propagated to all other cardiac phases using the cardiac displacement data inherently measured by cine DENSE imaging [38]. After segmentation, with the exception of manually identifying the right ventricular insertion point, the valve plane, and the apex, the remainder of the analysis was completely automatic.

For automatic phase unwrapping, the spatiotemporal guided-floodfill algorithm developed for 2D cine DENSE [39] was extended to three spatial dimensions plus time, and was applied to the voxels of the segmented myocardium. The unwrapped phase data were converted to Eulerian displacement by dividing by the displacement encoding frequency, and the 3D Eulerian displacement of each voxel was computed by means of vector addition of the three orthogonal one-dimensional (1D) displacement data.

Next, to convert 3D Eulerian displacements to 3D Lagrangian displacement trajectories, tissue tracking algorithms previously described for 2D data [39] were extended to 3D. Scattered data interpolation using radial basis functions of linear splines was performed to calculate the 3D motion trajectories of LV voxel centers identified at the initial acquired cardiac phase [40]. These trajectories were then slightly smoothed using 10th order polynomial functions.

A 3D strain analysis algorithm was also implemented. Specifically, each voxel of interest (VOI) at the initial displacement encoding moment was recorded as a position vector $\vec{u_{0}}$ in the *n*-dimensional space *R^n ^*(*n *= 3 in this 3D DENSE study):

(1) $$\vec{u_{0}}={\left[\begin{array}{ccc} u_{0,1} & \cdots & u_{0,n} \end{array}\right]}^{T}$$

where *u*_0,*j *_(*j *= 1, ..., *n*) corresponded to the coordinate component of $\vec{u_{0}}$, and the superscript *T *denotes the matrix transpose operation. *N *nearest available neighbor voxel position vectors (*N *≤ 12) were then identified in the closest three partitions within the myocardial contours for this VOI, and denoted as *n*-dimensional vectors $\vec{p_{0,i}}$ (*i *= 1, ..., *N*). The distance vector matrix *V_0 _*at the initial displacement encoding moment was formed by assembling the distance vectors pointing from the VOI to its *N *neighbors, as shown in Equation [2]:

(2) $$V_{0}=\left[\begin{array}{ccc} \vec{q_{0,1}} & \cdots & \vec{q_{0,N}} \end{array}\right]\;$$

where *V*_0 _was an *n *× *N *matrix containing the distance vectors $\vec{q_{0,i}}$ before deformation (defined in Equation [3]) in columns.

(3) $$\vec{q_{0,i}}=\vec{p_{0,i}}-\vec{u_{0}}\;\left(i=1,\cdots ,N\right)$$

At any certain cardiac phase *f*, the deformed distance vectors were denoted as $\vec{q_{f,i}}$, and the corresponding deformed distance vectors are expressed by *V_f _*in Equation [4]:

(4) $$V_{f}=\left[\begin{array}{ccc} \vec{q_{f,1}} & \cdots & \vec{q_{f,N}} \end{array}\right]\;$$

Assuming uniform and isotropic strain within the space specified by the VOI and its neighbors, the relationship between *V*_0 _and *V_f _*can be expressed by

(5) $$V_{f}=F_{f}V_{0}$$

where *F_f _*is the 3D deformation gradient tensor, an *n *× *n *matrix. In order to obtain a robust solution in the ill-conditioned case, singular value decomposition (SVD) was used to solve *F_f_*. Specifically, SVD was performed on $V_{0}V_{0}^{T}$, yielding the diagonal matrix *S *and unitary matrices *U *and *V *as shown in Equation [6]:

(6) $$V_{0}V_{0}^{T}=USV^{T}$$

Then another intermediate matrix *S*^0 ^was calculated as:

(7) $$\left\{\begin{array}{c} \begin{array}{cc} S_{i,k}^{0}=S_{i,k} & \;when\;i\neq k \end{array}\\ \begin{array}{cc} S_{i,i}^{0}=1∕S_{i,i}\;\;\; & else \end{array} \end{array}\right)$$

**Figure 2:**
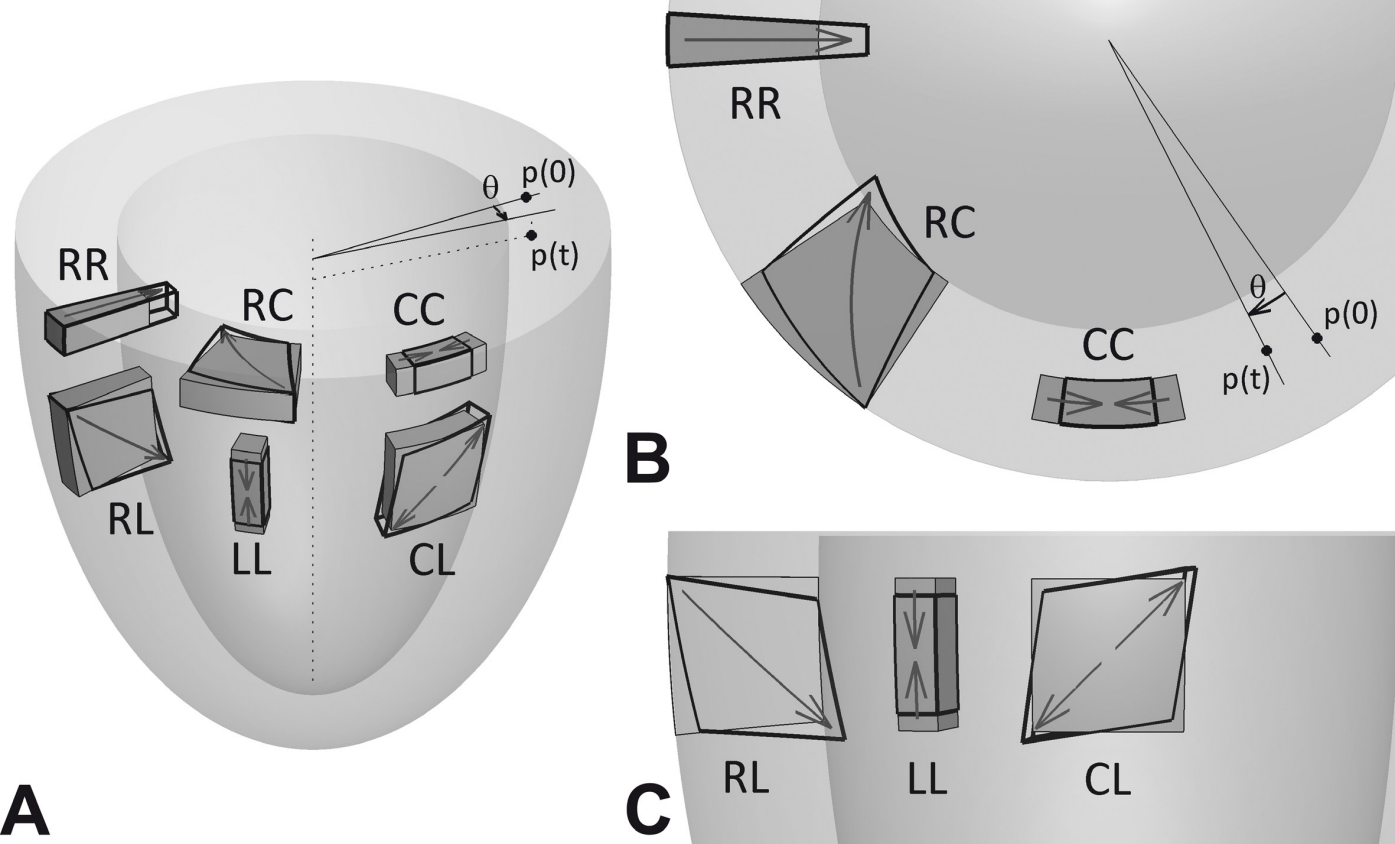
**Diagram of left-ventricular strain and twist**. Diagram of left-ventricular strain and twist. A 3D view is shown in (A), a 2D short-axis view as observed from the LV base is shown in (B), and a 2D long-axis view is displayed in (C). Strain measures the lengthening or shortening of muscle tissue in a given direction. During contraction, normal tissue lengthens radially (RR), shortens both circumferentially (CC) and longitudinally (LL), and shears slightly (RC, RL, CL). Twist (*θ*) measures the angular displacement of muscle tissue around the left ventricular centerline. During contraction, normal tissue undergoes a clockwise twist from p(0) to p(t).

Finally, the deformation gradient tensor *F_f _*was calculated as:

(8) $$F_{f}=V_{f}V_{0}^{T}VS^{0}U^{T}$$

and the 3D Lagrangian finite strain tensor *E_f _*was calculated as

(9) $$E_{f}=\left(F_{f}^{T}F_{f}-I\right)∕2$$

where *I *is the identity matrix. The strain tensor, *E_f_*, was decomposed into the local radial, circumferential and longitudinal (RCL) coordinate system, defined with respect to the LV contours at the first cardiac phase [41], to obtain the normal strains *E_rr_*, *E_cc_*, and *E_ll_*, and the shear strains *E_rc_*, *E_rl_*, and *E_cl _*[34]. The definitions of the normal and shear strains are illustrated in Figure 2.

The twist angle of a voxel was defined as the angle *θ *between the radial lines connecting the center of the slice to the center of the voxel of interest at end diastole (or the initial displacement encoding moment) and at each cardiac phase (Figure 2A and 2B). A positive angle was defined as a clockwise rotation when viewed from the base of the LV (Figure 2B). Torsion, defined as the normalized gradient of twist in the longitudinal direction, was calculated using Equation [10]:

(10) $$Torsion=\frac{\left(\theta_{apex}-\theta_{base}\right)R_{LV}}{L_{LV}}$$

where *θ_apex _*and *θ_base _*are the twist angles of the apex and the base of the LV, respectively, *R_LV _*is the average short-axis radius of the LV, and *L_LV _*is the long-axis length of the LV. This torsion definition is independent of ventricle size, and is well-suited for comparing torsion across mammals of vastly different sizes [42].

### Acquisition of 3D Cine DENSE Images in Mice

Seven wild type (WT) C57BL/6 mice (Jackson Laboratory, Bar Harbor, Maine, USA) were studied under protocols that conformed to the Guide for the Care and Use of Laboratory Animals (NIH publication no. 85-23, revised 1996) and were approved by the Animal Care and Use Committee at the University of Virginia. All imaging was performed on a 7T ClinScan CMR system (Bruker, Ettlingen, Germany) equipped with a gradient system that has a maximum strength of 650 mT/m and a maximum slew rate of 6666.7 mT·m^-1^·ms^-1^. For RF transmission and reception, a birdcage RF coil was used that has a diameter of 32 mm and an active length of 28 mm. During CMR, the mice were prone within the scanner, and heart rate, respiration, and core body temperature were monitored using a fiber optic, MR compatible system (Small Animal Imaging Inc., Stony Brook, NY, USA). Mouse body temperature was maintained at 36.4 ± 0.3°C during imaging using circulating thermostated water, and anesthesia was maintained using 1.25% isoflurane in O_2 _inhaled through a nose cone. Each complete CMR study took approximately 1 hour, including animal preparation, localizer scanning, and functional imaging.

For 3D cine DENSE CMR, pulse sequence parameters included voxel size = 0.25 × 0.25 × 0.4 mm^3^, TR = 7 ms, TE = 0.69 ms, flip angle = 15°, number of cardiac phases = 14, number of averages = 3, number of spiral interleaves = 27, number of spiral interleaves per cardiac phase per heartbeat = 1, and displacement encoding frequency = 1.1 cycles/mm. To achieve a balance of minimal off-resonance-induced blurring and data acquisition efficiency, the spiral readout duration was limited to 3 ms. The specific value of 3 ms was chosen based on the range of off-resonance frequencies that was recently measured in the mouse heart at a field strength of 7T [43]. Specifically, off-resonance frequencies in the range of ± 250 Hz occur under these conditions [43], which is an approximately 5-fold greater range than in the human heart at 1.5T [44]. Since a spiral readout duration of approximately 15 ms is typically used for human cardiac imaging at 1.5T [34], we reasoned that a readout duration reduced by a factor of 5 would provide a reasonable balance between scan efficiency and off-resonance-induced blurring under conditions found in the mouse heart. The field of view was 32 × 32 × 8.4 mm^3^, which covered the entire mouse LV, as the longitudinal dimension of the mouse LV is approximately 6 mm and the average radius of the mouse LV is approximately 2 mm. The scan time was approximately 23 minutes, depending on heart and respiratory rates. The imaging volume was oriented such that the 3D partition direction was aligned with the long axis of the LV. Fourteen 3D partitions were acquired symmetrically about the *k*-space origin, and zero-padding to 28 partitions was performed during reconstruction. After Fourier Transform in the partition direction, 3 partitions at each end of the volume were discarded to avoid aliasing. The resulting 3D image matrix size was 128 × 128 × 22.

## Results

**Figure 3:**
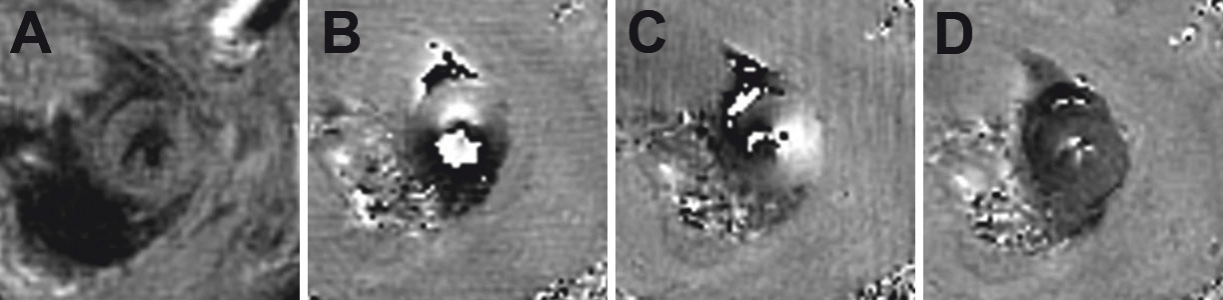
**Example end-systolic images from one mid-ventricular short-axis partition**. Example end-systolic images from one mid-ventricular short-axis partition from a 3D cine DENSE dataset of a mouse heart. A magnitude-reconstructed image is shown in (A), and phase-reconstructed images are shown in (B-D), where the image in (B) is encoded for displacement in the y-direction, the image in (C) is encoded for displacement in the x-direction, and the image in (D) is encoded for displacement in the z-direction. Phase wrapping within the myocardium occurred in (B) and (C), and is accounted for by phase unwrapping during image analysis.

**Figure 4:**
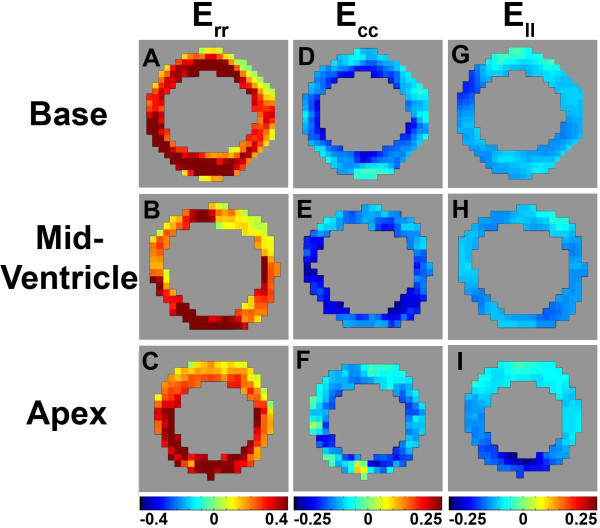
**Example end-systolic short-axis *E_rr_*, *E_cc_*, and *E_ll _*strain maps**. Example end-systolic short-axis *E_rr_*, *E_cc_*, and *E_ll _*strain maps at basal, mid-ventricular, and apical locations measured using 3D cine DENSE in a normal mouse. Fairly uniform shortening is observed for the circumferential and longitudinal strains, while fairly uniform lengthening is observed for radial strain.

**Figure 5:**
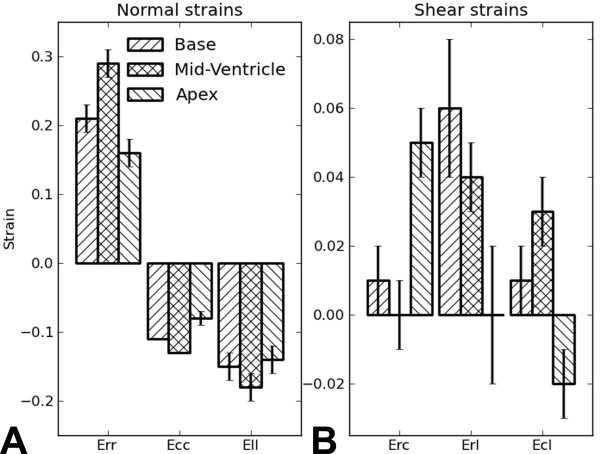
**Bar chart of the average end-systolic normal and shear strains. ** Bar chart of the average end-systolic normal (A) and shear strains (B) at the basal, mid-ventricular, and apical levels for the 7 healthy mice measured by 3D cine DENSE in this study. Data are plotted as mean ± standard error.

Image quality was routinely very good in the mid-ventricular region where off-resonance effects are usually minimal. An example demonstrating this image quality is shown in Figure 3, where a magnitude-reconstructed image is displayed as are phase-reconstructed images encoded for in-plane and through-plane displacement. Image blurring was more prevalent mainly toward the apex where off-resonance effects are greater. Nonetheless, image quality was adequate throughout most of the LV to achieve measurements of tissue displacement and myocardial mechanics. For example, maps of the end-systolic normal strains *E_rr_*, *E_cc_*, and *E_ll _*are shown in Figure 4 at basal, mid-ventricular, and apical slices. Figure 5 shows a bar chart of the average end-systolic strains at the basal, mid-ventricular, and apical levels. Heterogeneity in myocardial strains at different levels was observed (Figure 5), which is consistent with prior studies [22-25,32].

**Figure 6:**
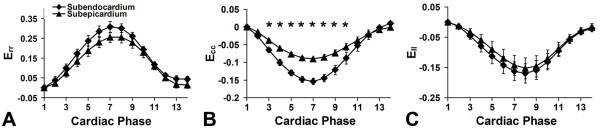
**Normal strain-time curves for healthy mice measured by 3D cine DENSE**. Normal strain-time curves for healthy mice measured by 3D cine DENSE. The average *E_rr_*, *E_cc_*, and *E_ll _*values for the 7 mice studied are plotted as a function of cardiac phase. Furthermore, separate curves are shown for the subendocardial and subepicardial layers. A statistically significant difference in *E_cc _*between the two layers was detected (B). No significant differences between layers were found for *E_rr _*and *E_ll_*, although both of these strains showed consistent trends towards larger absolute values in the subendo- vs. subepicardium. Data are plotted as mean ± standard error.

Using 3D cine DENSE, in addition to extensive spatial coverage, myocardial mechanics are measured as a function of cardiac phase across the cardiac cycle. For example, data summarizing the time-varying normal strains at the mid-ventricular level for all seven mice are shown in Figure 6, where *E_rr_*, *E_cc_*, and *E_ll_*, are plotted. These results demonstrate the measurement of normal radial thickening, circumferential shortening, and longitudinal shortening in healthy mice. The high spatial resolution of strain provided by cine DENSE is evident in these measurements, as statistically significant transmural differences in *E_cc _*are resolved using this technique, where the thickness of the LV wall is approximately 1 mm.

**Figure 7:**
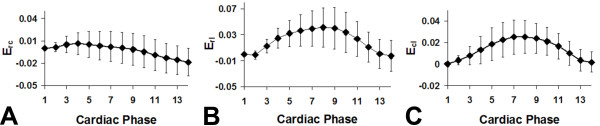
**Shear strain-time curves for healthy mice measured by 3D cine DENSE**. Shear strain-time curves for healthy mice measured by 3D cine DENSE. The average *E_rc_*, *E_rl_*, and *E_cl _*for the 7 mice are plotted as a function of cardiac phase. Data are plotted as mean ± standard error.

Using 3D cine DENSE, we were also able to measure the shear strains *E_rc_*, *E_rl_*, and *E_cl_*, as shown for the mid-ventricular region in Figure 7. As in humans, the shear strain magnitudes are lower than the normal strain magnitudes.

**Figure 8:**
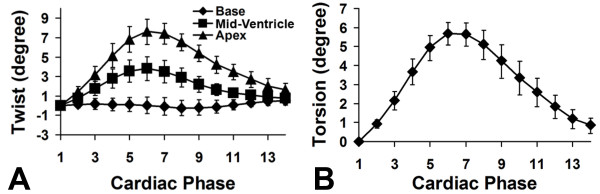
**Myocardial twist and torsion as a function of cardiac phase measured by 3D cine DENSE in seven mice**. Myocardial twist and torsion as a function of cardiac phase measured by 3D cine DENSE in seven mice. In (A), twist angle as a function of cardiac phase is shown for basal, mid-ventricular, and apical locations. In (B), LV torsion, which is the normalized gradient of twist in the longitudinal direction, is plotted. Data are plotted as mean ± standard error.

Lastly, using displacements that were measured in basal, mid-ventricular, and apical slices, we calculated myocardial twist and torsion as a function of cardiac phase. These results are displayed in Figure 8. Specifically, Figure 8A shows twist as a function of cardiac phase for short-axis slices at three different levels. At the basal level, very little twisting is observed. At the mid-ventricular level, an intermediate amount of counterclockwise twisting is seen, with a peak of approximately 4° at end systole. At the apical level, a greater amount of counterclockwise twisting is seen, with a peak of approximately 8° at end systole. In all slices, untwisting is observed during diastole. From twist data measured at multiple longitudinal levels, normalized LV torsion is computed as shown in Figure 8B, with a peak end-systolic value of 5.6 ± 0.9°.

## Discussion

The major contribution of this study is that a 3D cine DENSE method is described that enables a comprehensive evaluation of mouse heart mechanics with a scan time of less than 25 minutes and a data analysis time of approximately one hour. These times are significantly shorter than prior scan times of approximately one hour and prior image analysis times of several hours. The 3D cine DENSE method also provides relatively high spatial resolution, evidenced by the resolution of transmural differences in *E_cc _*and by the measurement of shear strains.

We used a 3D stack-of-spirals *k*-space trajectory to achieve efficient data sampling. To avoid excessive blurring due to off-resonance effects at 7T, we limited the spiral readout duration to 3 ms. We also used 3D field maps and previously described deblurring methods [37]. Using these techniques, image quality was good throughout the central 3.5 mm of the mouse heart. However, blurring was worse for approximately 1 mm at the base and 1.5 mm at the apex, where off-resonance effects were most prominent. In the future, shorter readouts and/or more sophisticated deblurring methods may improve image quality at these locations.

We measured peak normal end-systolic mid-ventricular strains in the mouse heart and found *E_rr _*values of 0.29 ± 0.17, *E_cc _*values of -0.13 ± 0.03 and *E_ll _*values of -0.18 ± 0.14. These values are in close agreement with prior 2D and 3D myocardial tagging studies [20,22-24], 2D HARP studies [25,26], and 2D DENSE studies [31-33]. As shown in Figure 5, we also measured the shear strains in the mouse heart. The systolic values of the shear strains are similar to the results of other shear strain measurements in mice [23]. Also, the shapes of the shear strain curves agree closely with other shear strain measurements in humans [34,45,46]. Finally, we computed twist at multiple short-axis levels as well a cardiac torsion. Accounting for different torsion definitions, the twist and torsion values are consistent with those reported previously [21-24].

A limitation of this study is that another independent technique such as myocardial tagging was not used in the same mice to validate 3D cine DENSE measurements. However, the literature contains 3D strain and torsion data for the mouse heart, and our results are similar to the literature values. Also, 3D cine DENSE was directly validated vs. 2D methods for human imaging [34], and this agreement increases confidence that the 3D cine DENSE data acquisition and analysis methods provide accurate data. Another limitation of the present study is that the image segmentation algorithms that we used were designed for 2D data, and were used separately for each longitudinal partition. A segmentation algorithm that identifies the 3D endocardial and epicardial surfaces will likely be faster and more accurate.

CMR of myocardial mechanics in mice enables the investigation of the roles of individual genes and experimental therapies in cardiac function. For example, Gilson et al and Vandsburger et al have used myocardial tagging in mice to elucidate the roles of genes that encode for nitric oxide synthases in myocardial mechanics [47,48]. HARP was recently used by Chuang et al to calculate 3D cardiac wall strain distributions from multi-slice tagging data in a genetically engineered mouse model of dilated cardiomyopathy [49]. In addition, PC imaging was applied by Nahrendorf et al to address pathophysiological issues in mice with a combined knockout of the mitochondrial and cytosolic creatine kinase (CK^-/-^) [28]. In a recent study, 2D cine DENSE was also applied to study the role of neuronal nitric oxide synthase in excitation-contraction coupling in the mouse heart [50]. Compared to the other methods, 3D cine DENSE provides the advantages of greater spatial coverage, high spatial and temporal resolution, and rapid post-processing. The 3D cine DENSE technique described in this study has great potential for evaluating the effects of experimental therapies and for quantifying the functional phenotype of genetically-engineered mice.

## Conclusions

Using a 3D cine DENSE sequence tailored for cardiac imaging in mice at 7T, a comprehensive assessment of 3D myocardial mechanics can be achieved with a scan time of less than 25 minutes and an image analysis time of approximately 1 hour.

## Competing interests

XZ is an employee of Siemens Medical Solutions USA, Inc. FHE received research funding from Siemens Medical Solutions.

## Authors' contributions

XZ and FHE participated in the design and development of the pulse sequence and post-processing software, study design, manuscript drafting and figure preparation. LBG performed data acquisition and analysis, and participated in figure preparation. BSS participated in the development of post-processing software. ADG participated in the development of post-processing software and figure preparation. CHM contributed to pulse sequence development, providing expertise in spiral imaging. BAF contributed expertise in cardiac physiology and participated in manuscript preparation. All authors read and approved the final manuscript.
